# Urinary Diversion and Metabolic Acidosis: A Case Report

**DOI:** 10.7759/cureus.14638

**Published:** 2021-04-22

**Authors:** Landric B Dsouza, Rana Jaffer H Hussein

**Affiliations:** 1 Emergency Department, Hamad Medical Corporation, Doha, QAT

**Keywords:** urinary diversion, cystectomy, metabolic acidosis

## Abstract

Urinary diversion, a surgical technique to redirect urine usually after cystectomy, and its complications are potential challenges to physicians and early recognition decreases mortality and morbidity. A 45-year-old male with a history of type 2 diabetes mellitus and hypertension for over three years underwent urinary diversion as a treatment for invasive bladder cancer and was discharged ambulatory with an indwelling Foleys catheter. The patient returned to the emergency room with a primary complaint of hematuria. The patient was noted to be frail-looking, dehydrated and tachycardic. Laboratory results yielded an acute kidney injury reflected by the elevated creatinine, hyperkalemia and a venous blood gas demonstrating a hyperchloremic metabolic acidosis. The patient had bilateral distended renal calyces by ultrasound and irrigation of bladder through a three-way Foley showed to have large thick clots. The patient was admitted under the surgical intensive care unit after involving appropriate sub-specialties. The patient was started on sodium bicarbonate infusion, broad-spectrum antibiotics and a urinary catheter in place to monitor urine output. The patient’s acidosis steadily improved with correction of his laboratory parameters, transferred out of ICU and the remaining stay in the medical ward was uneventful. The purpose of this case report is to help demonstrate the association between urinary diversion and the type of acidosis that subsequently develops in relation to this surgical procedure.

## Introduction

Urinary diversion, a surgical technique to redirect urine usually after cystectomy, and its complications are potential challenges to physicians and early recognition decreases mortality and morbidity. Urologic malignancies and genito-urinary tract abnormalities are frequent indications for this operative procedure; however, urinary diversions are associated with frequent metabolic derangements that pose challenges to the physician unfamiliar with the orthotopic neobladder. Emergency physicians play an important role in evaluating and diagnosing these complications and it is important to be aware of the spectrum of metabolic complications associated with this procedure.

## Case presentation

A 45-year-old male underwent nerve-sparing radical cystectomy with a urinary diversion in the form of Studer pouch as treatment of muscle-invasive bladder cancer. The neobladder was formed with an isolated segment of ileum with a urethral-ileal anastomosis.

Post-operatively, the patient’s hospital course was complicated with an extended-spectrum beta-lactamase (ESBL) urinary tract infection (UTI) and contrast computed tomography (CT) enhanced collection in the inguinal region, managed by drainage and antibiotic treatment under infectious disease stewardship and was discharged ambulatory with an indwelling Foleys catheter.

The patient returned three days after being discharged and was seen in the emergency room (ER) with a primary complaint of hematuria. The patient was noted to have a positive medical history of type 2 diabetes mellitus and hypertension for approximately three years. He was found to be frail-looking, dehydrated with dry mucous membranes, he was tachycardic (120 beats per minute), normotensive and afebrile. On abdominal examination, he was found to have a non-tender abdomen and a healing incision scar, with no signs of inflammation or infection. Laboratory results yielded a drop in hemoglobin, an acute kidney injury reflected by the elevated creatinine, hyperkalemia and a venous blood gas demonstrating a hyperchloremic metabolic acidosis (Table 1). The patient had bilateral distended renal calyces by bedside Point-of-Care Ultrasound (POCUS) and irrigation of the bladder through a three-way Foleys showed to have large thick clots. The electrocardiogram demonstrated sinus tachycardia.

**Table 1:** Blood Gas Analysis mmHg: Millimeters of mercury, mmol/L: Millimoles per liter, pH: Potential of hydrogen, pO_2_: Partial pressure of venous oxygen, pCO_2_: Partial pressure of venous carbon dioxide, Na: Sodium ion, K: Potassium ion, Cl: Chloride ion, HCO_3_: Bicarbonate ion

Blood Gas (Venous)	At Presentation	2 Hours After Presentation	Reference Range
pH	7.12	7.15	7.35–7.45
pO_2_ (mmHg)	<40	<40	35–45
pCO_2_ (mmHg)	34.8	35.4	30–40
Na (mmol/L)	132	133	135–145
K (mmol/L)	6.0	5.4	3.5–5.0
Cl (mmol/L)	110	113	96–110
HCO_3_ (mmol/L)	12	11	22–27
Base Excess	-16.4	-16.7	-2.0–2.0

The provisional diagnosis in the ER was thought to be urosepsis considering the patient’s recent UTI, also ureterolithiasis or intra-abdominal hematoma. On reviewing the earlier discharge summary, no expected complications, apart from the UTI, were documented following the surgical intervention. The urologist was then involved early for their opinion considering their expertise with the patient’s surgical history and was successively informed of the patient’s deterioration, persistent tachycardia and acidosis despite adequate fluid resuscitation and antibiotics in line with treatment for suspected sepsis.

The patient was admitted under the surgical intensive care unit (SICU) and was worked by nephrology for pre-renal medical causes and post-renal obstruction as a reason for the deranged renal parameters. The patient was started on sodium bicarbonate infusion, broad-spectrum antibiotics and a urinary catheter in place to monitor the urine output. The acidosis steadily improved with correction of the laboratory parameters, the patient was transferred out of ICU and the remaining stay in the medical ward was uneventful. He was eventually discharged without a Foleys catheter, with voluntary voiding of urine and with a follow-up appointment to urology.

## Discussion

Invasive bladder cancer is a commonly encountered urological malignancy where the standard of care is a surgical treatment in the form of radical cystectomy with urinary tract reconstruction. Although there are several methods offered to patients, since the 1980s orthotopic neobladder, using intestinal tissue to create a urinary diversion to the intact native urethra has found to be favorable [1].

Neobladders have added quality of life to patients who suffer from invasive bladder cancer but at the cost of certain complications, particularly metabolic abnormalities which have been frequently reported in the literature [2,3]. The main cause seems to stem from the increased absorption of ammonium ions through the intestinal mucosa with severity depending on the type of intestinal tissue used in creating the conduit and the duration of urine exposure to the intestinal tissue [2,4]. Additionally, pre-existing co-morbidities play a role in exacerbating the acidosis due to failure of compensation of the metabolic acidosis.

Hyperchloremic metabolic acidosis develops when the bowel segment absorbs ammonia, hydrogen and chloride ions in exchange for the excretion of the sodium and bicarbonate ions. The early post-operative period has been shown to demonstrate a mild metabolic acidosis in up to 70% of patients; however, the impaired renal function raises the risk of developing persistent metabolic acidosis due to the inability to compensate the metabolic abnormalities [5,6].

In our patient, his presenting symptoms were suggestive of a post-renal obstruction due to blood clots which possibly resulted in exacerbating his persistent metabolic acidosis. A narrow differential exists for normal anion gap metabolic acidosis constituting renal tubular acidosis, severe diarrhea and complications of urinary diversions. The initial diagnosis of normal anion gap metabolic acidosis due to urinary diversion may have been considered but due to unfamiliarity with the surgical operation, its expected complications, and the patient’s recent UTI and reduction in his hemoglobin, alternative diagnoses were pursued in the emergency setting.

Involving respective sub-specialties in the management of this patient led to treatment with intravenous sodium bicarbonate in the correction of the acidosis, which is the mainstay of treatment of severely low pH. However, if metabolic acidosis is predicted to occur, oral alkalizing agents such as sodium bicarbonate may be offered early to patients as even if asymptomatic, chronic metabolic acidosis affect bone growth and structure due to demineralization [7,8].

## Conclusions

This case highlights the importance of recognizing hyperchloremic metabolic acidosis in patients with surgical urinary diversions. A delay in proper treatment and early sub-specialty involvement can result in significant morbidity. Educational points include a clear discharge summary from sub-specialties of the surgical procedures offered and the expected complications to be aware of in case of a return visit to the ER. 
